# Mosaic derivative chromosomes at chorionic villi (CV) sampling are expression of genomic instability and precursors of cryptic disease-causing rearrangements: report of further four cases

**DOI:** 10.1186/s13039-024-00675-3

**Published:** 2024-04-08

**Authors:** Giulia Vitetta, Laura Desiderio, Ilaria Baccolini, Vera Uliana, Giulia Lanzoni, Tullio Ghi, Gianluigi Pilu, Enrico Ambrosini, Patrizia Caggiati, Valeria Barili, Anna Carmela Trotta, Maria Rosaria Liuti, Elisabetta Malpezzi, Maria Carla Pittalis, Antonio Percesepe

**Affiliations:** 1 Medical Genetics Unit, IRCCS Azienda Ospedaliero-Universitaria di Bologna, Bologna, Italy; 2 Medical Genetics Unit, University Hospital of Parma, Parma, Italy; 3 Obstetrics & Gynecology, Department of Medicine and Surgery, University of Parma, Parma, Italy; 4 Obstetric Unit, IRCCS Azienda Ospedaliero-Universitaria di Bologna, Bologna, Italy; 5 Medical Genetics, Department of Medicine and Surgery, University of Parma, Parma, Italy; 6 TOMA Advanced Biomedical Assays S.p.A, Busto Arsizio, Varese Italy

**Keywords:** Chorionic villi, Genomic instability, Cryptic rearrangement

## Abstract

Mosaic chromosomal anomalies arising in the product of conception and the final fetal chromosomal arrangement are expression of complex biological mechanisms. The rescue of unbalanced chromosome with selection of the most viable cell line/s in the embryo and the unfavourable imbalances in placental tissues was documented in our previous paper and in the literature. We report four additional cases with mosaic derivative chromosomes in different feto-placental tissues, further showing the instability of an intermediate gross imbalance as a frequent mechanism of de novo cryptic deletions and duplications. In conclusion we underline how the extensive remodeling of unbalanced chromosomes in placental tissues represents the ‘backstage’ of de novo structural rearrangements, as the early phases of a long selection process that the genome undergo during embryogenesis.

## Introduction

Meiotic and somatic chromosomal mosaicisms always result from a post-zygotic error leading to rescue or de novo origin of the new cell line, respectively. Different factors, i.e. timing and position of the mutated cell together with active or passive cellular competition, may result in different mosaic patterns with non-homogeneous distribution along the cytotrophoblast-extraembryonic mesoderm-embryo axis [2, 14]. Moreover, the structural instability of genome during human cleavage stage embryogenesis is currently well known [22, 28], under the hypothesis that cell division control mechanisms relax in zygote segmentation to allow for the rapid expansion of the conceptus, leading to several numerical and complex structural chromosomal anomalies on which repair mechanisms act to recover a viable embryo [1]. In our previous paper we documented three prenatal cases in which confined placental gross unbalanced rearrangements were the marker of cryptic fetal terminal deletions, suggesting the role of placental tissue analysis in their diagnosis and the better tolerance of placenta towards larger imbalances, with selection of smaller ones in the embryo [21]. Early embryonic developmental bottlenecks have been suggested selecting cytogenetically abnormal cells from the inner cell mass resulting in confined placental mosaicism [4]. Recently, Zuffardi and colleagues [34] speculated and discussed “the embryo battle against adverse genomes”, which finally results in viable terminal deletions in the embryo and to non-viable imbalances confined to placenta. We report four additional cases with mosaic derivative chromosomes in different feto-placental tissues, further documenting the instability of an intermediate gross imbalance as a frequent mechanism of de novo deletions and duplications.

## Methods

Cytogenetic analysis was performed on prenatal and postnatal cells according to our laboratory’s routine procedures [24]. Chromosomes were processed by G- and/or Q-banding techniques and analyzed at a resolution of 300 bands per haploid set (ISCN, 2020) for CV direct preparation, at least 400 bands for CV long-term culture preparation, and up to 550 bands for amniocytes and lymphocytes. FISH analysis was performed on metaphase spreads using whole-chromosome paint (Technogenetics/Kreatech) and subtelomeric (Vysis/Kreatech) probes in accordance with the respective manufacturers’ protocols. DNA was extracted from cultured and uncultured placental cells and amniocytes using the QIAamp DNA Tissue Mini Kit (Qiagen) in accordance with the manufacturer’s instruction. Chromosomal microarray analysis (CMA) was performed using Agilent Technologies 8 × 60K International Standard Cytogenomics Array (ISCA) Consortium configuration (resolution of about 75 kb). Slides were scanned using a G2539A Agilent microarray scanner (Agilent Technologies) and analysed using Cytogenomics (v5.0) microarray software. Genomic position refers to hg19 genomic Build.

## Clinical reports

### Patient 1

**Fig. 1:**
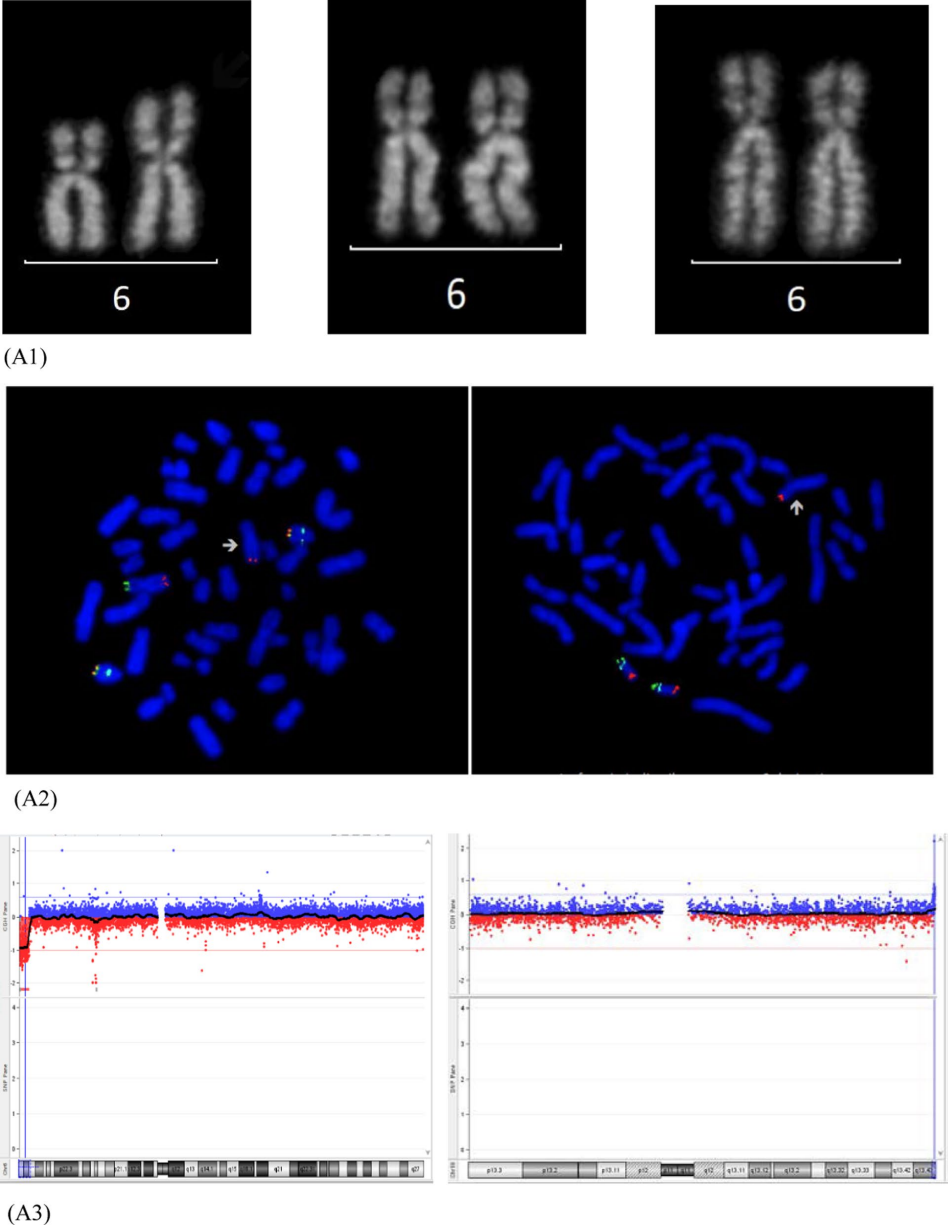

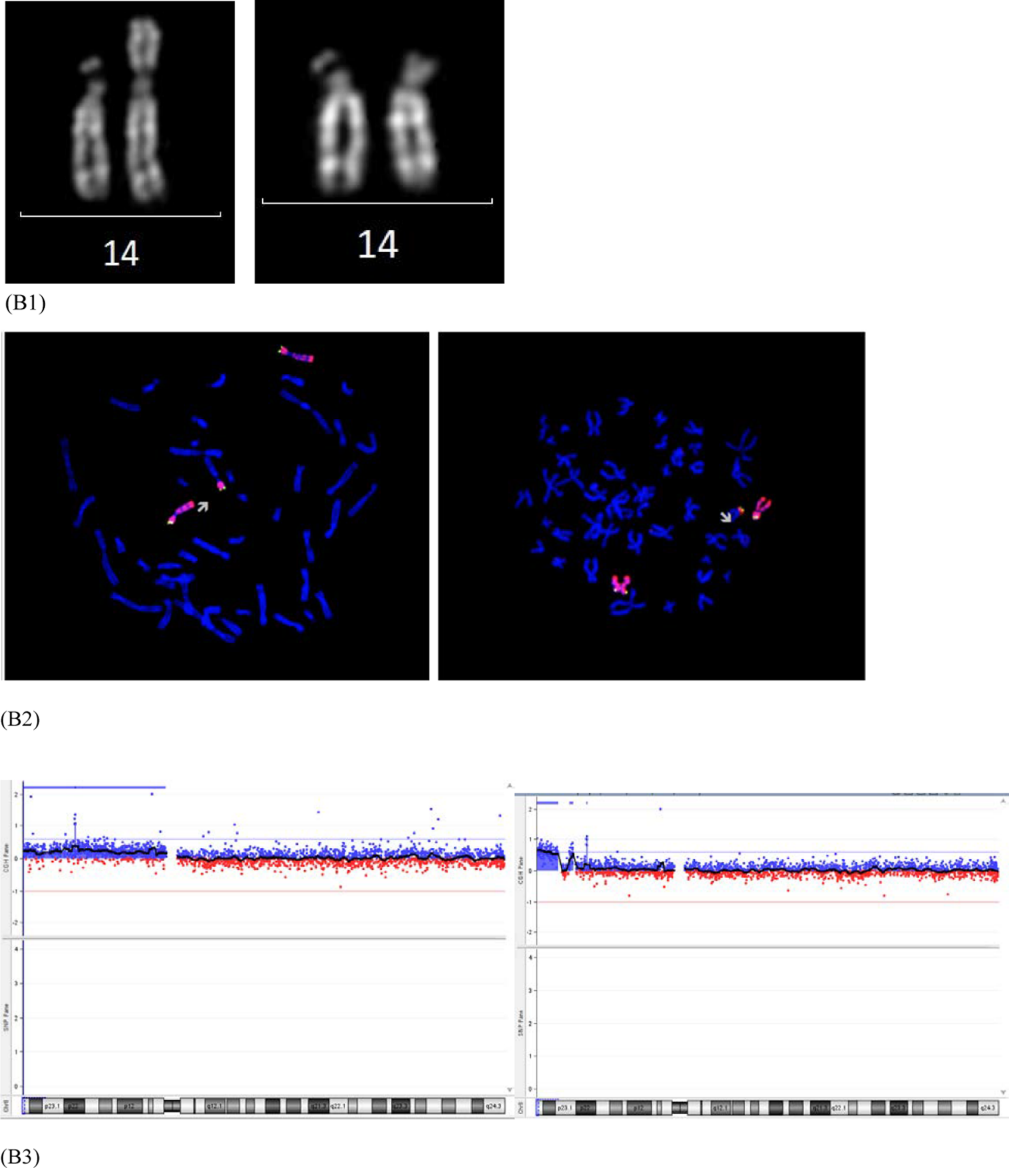

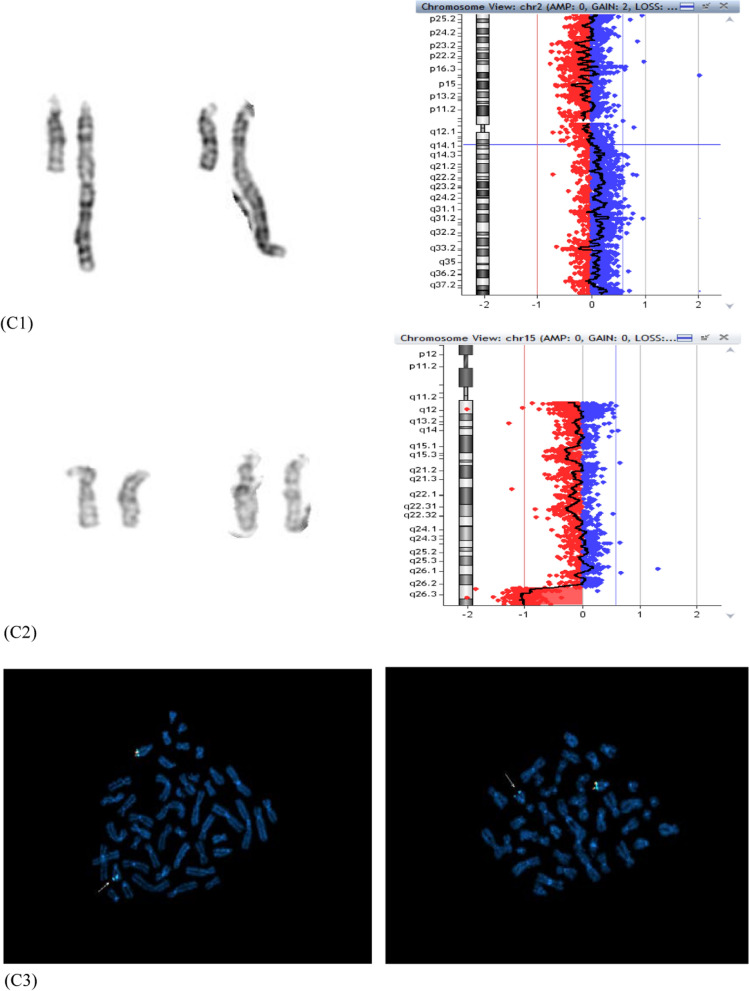
Simple unbalanced translocations. The arrows indicate the abnormal chromosomes. Case1, panel **A**: (A1) Representative pair of Q-banded add(6)(p25) in the cytotrophoblast (left) and cryptic der(6)t(6;19) in placental mesenchymal cells (center) and in amniocytes (right) (A2) Subtelomeric FISH on cryptic derivative: the green signal marks the 6p subtelomere, with cryptic terminal deletion in the derivative (left); the red signal marks the 19q subtelomere, with cryptic 19q duplication in the derivative (right) (A3) Cryptic derivative CMA profiles: chromosome 6 with 6p25 microdeletion (left) and chromosome 19 with 19q13.43 microduplication (right). Case 2, panel **B**: (B1) Representative pair of Q-banded chromosomes 14: the chromosome pair with the add(14)(p11.1) (left) and the apparently normal one (right) (B2) Subtelomeric and whole-chromosome 8 painting FISH on the two cell lines: the chromosome 8 is labeled in red, the green signal marks the 8p subtelomere in the cell line with the larger duplication 8p23.3p11.1 (left) and in the one with the smaller non adjacent duplications 8p23.3p23.1 and 8p23.1p23.1 (right) (B3) chromosome 8 CMA profile from uncultured CV, showing a mosaic duplication of the entire 8p (DNA from a mixture of normal cytotrophoblast and non-mosaic abnormal mesenchyme) (left) and from amniocytes of an independent culture with only the cryptic derivative (8) that shows two discontinuous non-mosaic duplications in 8p. Case 3, panel **C**: (C1) Representative pairs of G-banded chromosomes 15 with the der(15)t(2q;15q) on the right (left) and chromosome2 CMA profile from CV mesenchyme with mosaic 2q14.1q37.3 duplication (right) (C2) Representative pairs of G-banded apparently normal chromosomes 15 (left) with terminal non-mosaic microdeletion 15q26.2q26.3 in CMA profile (left) (C3) Subtelomeric FISH on apparently normal cell lines: terminal microdeletion 15q26.2q26.3 with loss of the subtelomeric orange/green signal distal to light blue control probe, in cells from CV mesenchymal cells (left) and from CV direct preparation (right)

A 34-year-old woman was referred to genetic counselling for trisomy 21 (T21) increased risk at the combined test. CV sampling was performed at 13th weeks’ gestation, showing a non-mosaic add(6)(p25) in 12 metaphases analysed from the direct preparation, with a normal karyotype in 22 cells from long-term culture. Karyotype was apparently normal in 20 metaphases from 10 colonies analysed from amniotic fluid, whereas CMA and FISH analysis showed a cryptic translocation derivative resulting in a 4.2 Mb deletion at 6p25.1 and a 300 kb duplication at 19q13.43 (Fig. 1, panel A). Parental karyotypes with FISH analysis were normal. The fetal karyotype was described as 46,XY.ish der(6)t(6;19)(p25.1;q13.43)(6PTEL48-,D19S238E+)dn.arr[GRCh37] 6p25.3p25.1(204009_4397431)×1,19q13.43(58800403_59095418)×3. A retrospective subtelomeric FISH analysis demonstrated the cryptic rearrangements also in the CV mesenchymal cells and confirmed the origin from chromosome 19q of additional material on add(6) in the CV trophoblastic cells together with the presence of the microdeletion 6p25.1.

The 4.2 Mb deletion is causative of the Chromosome 6pter-p24 Deletion Syndrome (OMIM #612582), characterized by variable degrees of intellectual disability and multiple malformations. The variant includes the FOXC1 gene (OMIM *601090), which in deletion causes the Axenfeld-Rieger syndrome type 3 (OMIM #602482), exhibiting a phenotypic overlap with 6pter-p24 deletion syndrome. The pre-morphological ultrasound performed at 16th weeks’ gestation revealed a slight imbalance of the cardiac chambers, with a prevalence of the right sections, and the prominence of the fourth ventricle of the posterior cranial fossa. The pregnancy was terminated at 18th weeks’ gestation. For future pregnancies, the couple has the choice to rule out the possibility of germline mosaicism (1% risk) through prenatal invasive procedure.

### Patient 2

A 31-year-old woman underwent villocentesis at 13th weeks’ gestation due to T21 increased risk at the combined test.

Direct CVS analysis showed a 46,XX karyotype in all metaphases, whereas the cultural CV analysis documented the presence of a non-mosaic add(14)(p11.1), characterized by CMA from uncultured CV as a duplication 8p23.3p11.1 of the entire short arm of a chromosome 8 [der(14)t(8;14)(p11.1;p11.1); about 43 Mb] (Fig. 1, panel B). The mosaic form of 8p23.3p11.1 duplication (log2 ratio of + 0.32) confirms the presence of a normal cell line in the cytotrophoblast. Karyotype, CMA and subtelomeric FISH analysis on amniocytes identified the previous derivative but in mosaic with an additional der(14) with a terminal 8p23.3p23.1 duplication (7 Mb) and an interstitial 8p23.1p23.1 duplication (1.3 Mb), close by and discontinuous. The chromosome 8 CMA profile on DNA from amniocytes (in an independent culture where the bigger derivative was absent in the few starting colonies) showed both the discontinuous duplications in non-mosaic form, suggesting that they were both on the same chromosome (Fig. 1, B3 right). Parental karyotypes and FISH analysis were normal. The fetal karyotype was described as mos 46,XX,der(14)t(8;14)(p11.1;p11.1)dn[32].ish der(14)(RH65733+,wcp8+).arr[GRCh37] 8p23.3p11.1(119720_43430652)×3/46,XX,der(14)t(8;14)(p23.1;p11.1)dup(8)(p23.1p23.1)[10].ish der(14)(RH65733+,wcp8+).arr[GRCh37] 8p23.3p23.1(194625_6911531)×3,8p23.1(10521995_11805960)×3.

The involvement of SOX7 (OMIM *612202) and GATA4 (OMIM *600576) in both duplications is responsible for the 8p23.1 Duplication Syndrome, with a highly variable phenotype, mainly characterized by developmental delay/intellectual disability, dysmorphic features and congenital heart disease. A second level morphological ultrasound was performed at 20th week’s gestation and showed agenesis of the corpus callosum associated with bilateral colpocephaly, deviation of the thoracic vertebral column, persistence of the right umbilical vein with aneurysmal dilatation in its proximal portion, interventricular septal defect with reduced aortic diameters and anterograde flow.

Considering even the high risk of syndromic intellectual disability associated with the abnormal ultrasound findings, the patient opted for termination of pregnancy. Regarding the recurrence risk, the 1% residual risk for germline mosaicism can be investigated in future pregnancies through invasive testing procedures.

### Patient 3

A 42-year-old woman underwent CV sampling at 13 weeks’ gestation because of increased nuchal translucency (6.9 mm). Direct preparation showed a normal karyotype in 10 metaphases, while a der(15)t(2q;15q) was observed in 10 of 40 cultured cells. The CMA performed on mesenchymal cells showed a mosaic duplication in 2q14.1q37.3 (129 Mb; log2 ratio of + 0.32) and a non-mosaic deletion in 15q26.2q26.3 (6.9 Mb), suggesting the cryptic deletion both in the apparently normal line and in the derivative chromosome. The microdeletion was indeed confirmed by subtelomeric FISH in the normal cell line both from culture and from direct preparations (Fig. 1, panel C). Parents’ karyotype with FISH analysis were normal. The karyotype was described as 46,XX.ish del(15)(q26.3)(D15S936-)dn in the direct preparation and mos 46,XX[30].arr[GRCh37] 15q26.2q26.3(95495059_102383473)×1 dn/46,XX,der(15)t(2;15)(q14.1;q26.2)dn[10].arr[GRCh37] 2q14.1q37.3(114317104_243068936)×3,15q26.2q26.3(95495059_102383473)×1 in the long-term culture.

The mosaic gross duplication in 2q14.1q37.3 (129 Mb) is most likely incompatible with a long embryo survival. The microdeletion 15q26.2q26.3 (6.9 Mb) is associated with Chromosome 15q26-qter Deletion Syndrome (OMIM #612626), characterized by growth restriction, variable degrees of intellectual disability and congenital anomalies, including heart malformations. Fetal nuchal translucency thickness is a common phenotypic expression of fetal heart anomalies [23]. Moreover, the deleted segment included NR2F2, a disease-causing gene (OMIM *107773) involved in angiogenesis and heart development, proposed as candidate for congenital cardiac defects, multiple types (OMIM #615779) (Benbouchta et al., [3]). The probable correlation of the placental chromosomal result with the fetal echographic sign led the patient to refuse the amniocentesis and to request termination of pregnancy. Consensus to the cytogenetic follow-up on the aborted fetus was denied.

### Patient 4

**Fig. 2:**
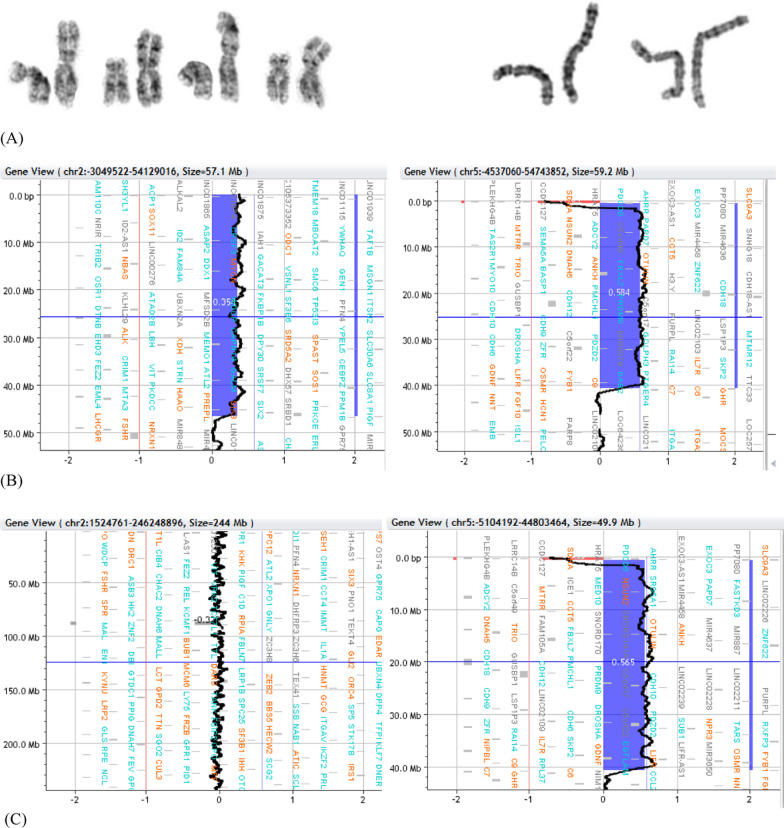
Inv-dup del translocation. **A** Representative pairs of G-banded chromosome 5 (derivative 5p on the right): inv-dup del t(2;5) derivative in direct preparation (left) and inv-dup del(5) derivative in long-term culture (right) **B **Inv-dup del t(2;5) CMA profiles in CV direct preparation, with mosaic duplication 2p25.3p21 (log2 ratio + 0.35) (left) and non-mosaic terminal microdeletion 5p15.33 adjacent to duplication 5p15.33p13.1 (right). **C** Inv-dup del(5) CMA profile in CV long-term culture, showing the absence of 2p25.3p21 duplication (a normal profile for chromosome 2 on the left) and the remaining non-mosaic derivative inv-dup del(5) (right)

A 29-year-old woman underwent CV sampling at 13 weeks’ gestation because of fetal cystic hygroma. Twenty cells from direct preparation showed an add(5)(p15.3), while 20 cells from long-term culture displayed a smaller add(5)(p15.3) (Fig. 2A). CMA performed on the two samples allowed the characterization of the two different derivatives as (i) uncultured CV: a translocation between chromosome 2p and chromosome 5p, presenting with a mosaic terminal duplication in 2p25.3p21 (47 Mb; log2 ratio of + 0.35), a non-mosaic terminal microdeletion in 5p15.33 (539 kb) with contiguous interstitial duplication in 5p15.33p13.1 (40 Mb) which might be inverted and correspond to the classical inv-dup del translocation (2p;5p) (Fig. 2B); (ii) cultured CV: the non-mosaic inv-dup del(5) without the translocated segment (Fig. 2C). The admixture of both genomes from cytotrophoblast and from mesenchyme explains the mosaic form of 2p25.3p21 duplication seen in the uncultured CV, duplication which however seems to be present in non-mosaic form in the cytotrophoblast and to be absent in the mesenchymal cells. Parents’ karyotypes with FISH analysis were normal, even if a parental microinversion cannot be excluded [12].

The final karyotype was described as: 46,XX,der(5)(2pter- > 2p21::5p15.33::5p13.1- > 5p15.33::5p15.33- > 5qter)dn.arr[GRCh37] 2p25.3p21(17019_46501071)×3[0.3],5p15.33(22149_560827)×1,5p15.33p13.1(574904_40759663)×3 and 46,XX,der(5)(:5p15.33::5p13.1- > 5p15.33::5p15.33- > 5qter)dn.arr[GRCh37].

5p15.33(22149_560827)×1,5p15.33p13.1(574904_40759663)×3, in the uncultured and cultured preparations, respectively.

Some cases of distal inv-dup del 5p have been reported, three of which were prenatally detected presenting abnormal ultrasound or autopsy findings [13, 18, 27], including one with cystic hygroma [27], as in our case. The fetal inv-dup del of Izzo and colleagues [13] is very similar to ours (870 kb terminal microdeletion adjacent to 40.5 Mb inverted duplication) with mild ultrasonographic anomalies. As in our case, the critical region for Cri-du-chat syndrome (5p15.2) was not included in the deletion, which however encompassed the 5p15.3 region, associated with microcephaly and cat-like cry features of the syndrome [9, 20]. The patient refused the proposed amniocentesis in order to verify the real fetal chromosomal arrangement, opting for termination of pregnancy on the basis of the fetal malformation and its probable correlation with placental abnormal karyotypes. Abortion was performed in a different obstetric unit, therefore not allowing a cytogenetic follow-up on the tissues of the aborted fetus.

## Discussion

Complex meiotic and post-zygotic mechanisms underlie non-mosaic or mosaic structural chromosomal anomalies [33]. De novo unbalanced translocations may arise from non-disjunction at the maternal meiosis I or II, followed by a trisomy rescue through anaphase lagging of the supernumerary chromosome: this in turn undergoes a chromothripsis event [16], with preservation of the telomeric fragment by joining to a recipient chromosome which has lost its terminal portion [5, 30]. Alternative mechanisms of deletion-induced repair pass through a meiotic (NAHR or U-type exchange) or mitotic pre-meiotic/post-zygotic (NHEJ) models leading to a simply deleted chromosome or to an intermediate dicentric chromosome, whose asymmetric breakage results into an inv-dup del and a complementary deletion. All the intermediate products are stabilized through telomere capture by another chromosome, leading to simple or complex unbalanced translocations [5]. An unstable meiotic or post-zygotic dicentric chromosome may also be the starting point for mosaic structural imbalances, followed by its meiotic or early post-zygotic breakage [26]. Chromosome instability is common in human cleavage-stage embryo. The possibility of consecutive breakage-fusion-breakage (BFB) cycles may lead to very complex derivatives of the same chromosome, with a selection of the most viable cell line(s) in the fetus and a confined placental mosaicism of the most severe imbalances [21, 25, 28].

**Fig. 3:**
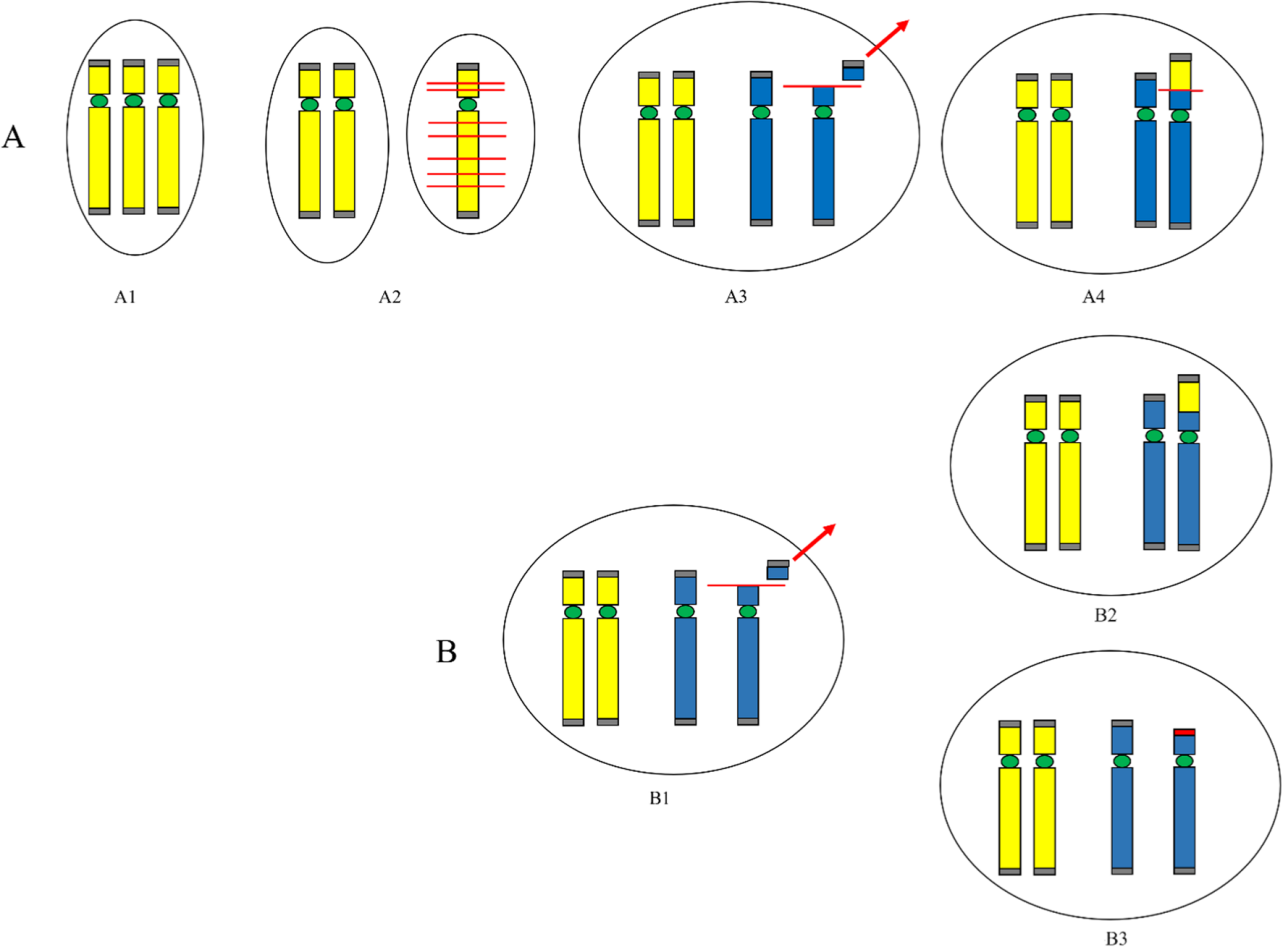
Schematic representation of mechanisms leading to gross simple unbalanced translocations in case 1, 2 and 3. Donor chromosome pair is in yellow, recipient chromosome pair is in blue, the telomeric sequences are in grey, the red line indicates the breakage event, the sticky terminal portion of deleted chromosome that will stabilize in different ways is indicated in red. Mechanism **A** (upper sequence): The trisomy (A1) normalizes by anaphase lagging with chromothripsis of the supernumerary chromosome in the micronucleus (A2, right); a breakage event in the recipient chromosome (either in p or in q arm) with loss of the terminal portion (A3), the deleted chromosome stabilizes by telomere (p or q) capture from the fragmented chromosome leading to the simple unbalanced translocation (A4). This mechanism presupposes a second chromosomal breakage event at the same initial breakpoint in the recipient chromosome (A4) for the onset of the second cryptic derivative chromosome, either by a second chromothripsis event and smaller segments recapture (case 1 and 2) or by loss of the translocated portion and neo-telomere formation (case 3) (see the text for the details). Mechanism **B** (lower sequence): a breakage event in the recipient chromosome (either in p or in q arm) with loss of the terminal portion leads to the deleted chromosome (B1) which stabilizes in different independent ways in the two daughter cells during early embryonic development, by telomere (p or q) capture from donor chromosome in one cell (B2, the result is the simple unbalanced translocation) and by de novo telomere synthesis in the other cell (B3, the result is the cryptic derivative)

Cases 1, 2 and 3 presented with simple unbalanced translocations (class A, [5]) visible at conventional cytogenetics in different placental tissues as markers of a cryptic non-mosaic (case 1) or mosaic (case 2) unbalanced translocation in the fetus, or of a cryptic terminal deletion in placental tissues (case 3). Different mechanisms can be considered to explain the three simple unbalanced translocations visible at karyotype, from a more intriguing initial trisomy rescue with a chromotripsis event and telomere capture, to a more straightforward very early post-zygotic error, with a rescue event by telomere capture in one daughter cell (Fig. 3, panel A). The trisomy rescue by anaphase lagging of the supernumerary chromosome (19, 8 and 2 in our cases), followed by its segregation within a micronucleus where shuttering occurs, is at the basis of many chromothripsis events [17, 31, 32], followed by the retrieval of its telomeric portion by a different recipient chromosome that loses its distal portion (6, 14 and 15 in our cases), thus forming the de novo unbalanced translocation with terminal deletion of the recipient chromosome and terminal duplication of the donor one [5, 30]. Chromosomal rearrangements involving chromosome 1, 2, 3, 5, 7, 8, 13,15, 19, X resulting from chromothripsis event were observed and characterized [7, 8, 15, 19, 29]. The donor chromosomes involved in our cases 1, 2 and 3 were chromosomes 19, 8 and 2, respectively, so the hypothesis for gross unbalanced translocations visible at conventional cytogenetics as result from a chromotripsis event might be realistic. In the mechanism A the onset of the additional cryptic derivatives observed in all the three cases has to consider a second independent post-zygotic break at the same locus as the initial break event, which might seem most unlikely, as expression of the intense post-zygotic remodeling aimed at recovering a viable embryo [6, 21, 26]. In particular, for case 1 we assume a further chromothripsis event leading to shattering of the additional 19q and recapture of the 19q13.43 portion containing the telomeric sequence. Concerning Case 2, the presence of a normal cell line in the cytotrophoblast indicates an early post-zygotic onset in the progenitor cell of the inner cell mass of the larger simple unbalanced translocation involving chromosome 8, with an incomplete rescue event leading to the second cryptic derivative with non-adjacent duplications. As in case 1, we have to think to a chromothripsis event with the shattering of the additional 8p, which would have been partially reattached to the derivative chromosome 14 (the 8p23.3p23.1 portion with telomeric sequence and the smaller interstitial 8p23.1p23.1 segment). Differently, for case 3 we have to consider the loss of the translocated 2q14.1q37.3 segment with stabilization of the deleted chromosome 15 by neo-telomere formation. The alternative more straightforward and perhaps more probable mechanism B, with a very early post-zygotic error followed by independent chromosome healing of both sister chromatids by telomere capture and de novo telomere synthesis in the two daughter cells explains the onset of both derivatives in case 1 and 3 (Fig. 3, panel B).

**Fig. 4:**
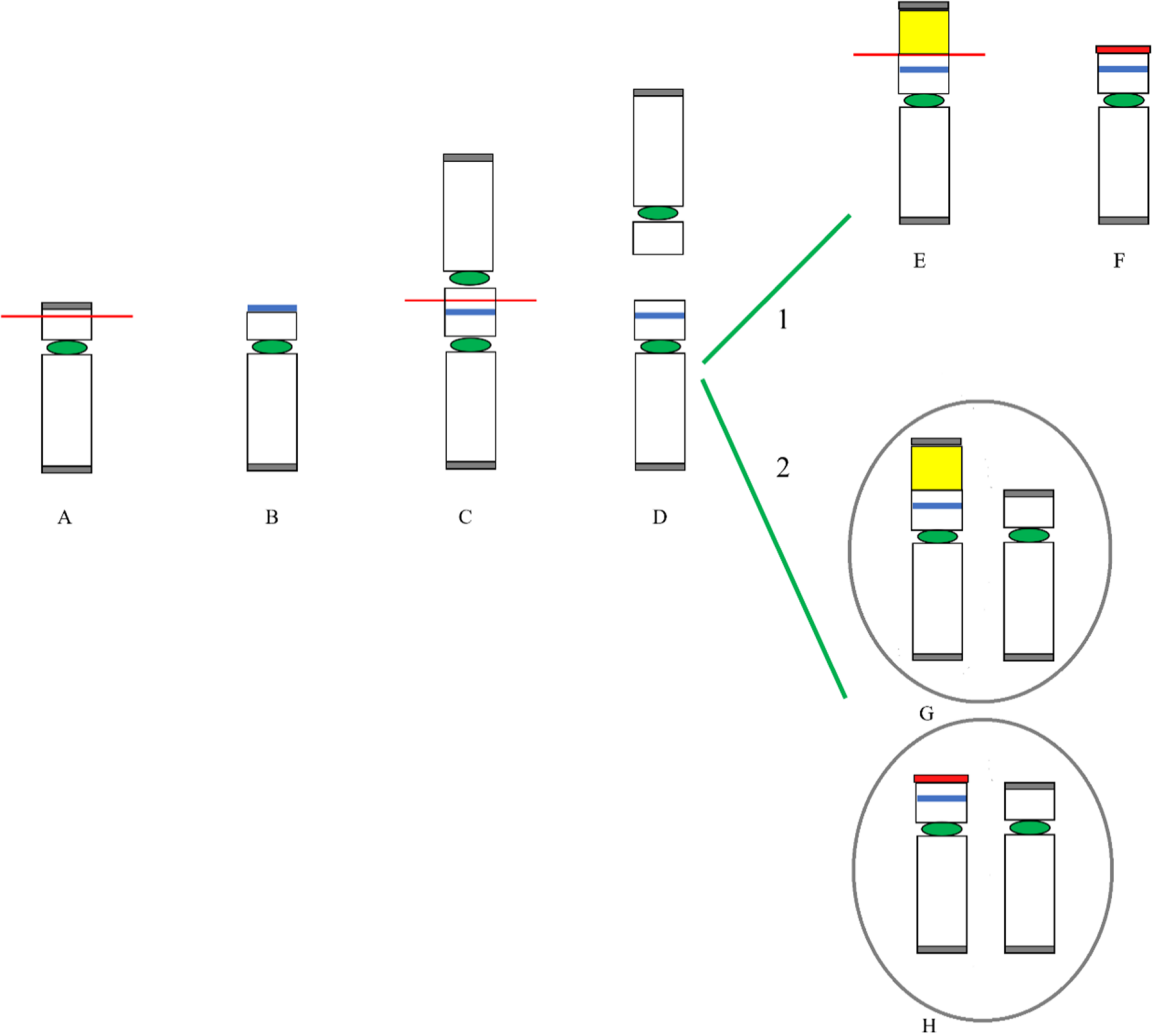
Schematic representation of mechanisms leading to inv-dup del translocation (2p;5p) and inv-dup del(5) in case 4. The red line indicates the breakage event, the deletion on chromosome 5 is in blue, the portion of donor chromosome 2 is in yellow, the telomeric sequences are in grey, the neo-telomere is in red. A breakage event on chromosome 5p **A** leads to the deleted chromosome **B** that stabilizes its sticky end by forming an unstable intermediate dicentric **C** whose asymmetric breakage results into an inv-dup del and a deleted chromosome **D**; the inv-dup del stabilizes by capturing a large portion of chromosome 2p containing the telomeric sequence, leading to the inv-dup del translocation (2p;5p) **E**, **G**. Two mechanism may be considered to explain also the presence of the smaller inv-dup del (5): the mechanism 1 (upper) presupposes the loss of the translocated chromosome 2 portion with stabilization of the inv-dup del (5) by de novo telomere synthesis **F**, the mechanism 2 assumes two independent repair events in the two daughter cells of the mitotic division, which stabilize the inv-dup del either by translocation with 2p in one cell daughter (**G** left) or by neo-telomere formation in the other ones (**H** left)

The case 4 showed a complex inv-dup del translocation (class B, [5]), with deletion and duplication which seemed adjacent at the resolution of the array used (inv-dup dels can present or not a single copy region between the two different imbalances). Although we could not perform any FISH experiment to test the orientation of the duplicated region, it seems likely that it was inverted, in analogy with similar reports with contiguous imbalances [13]. All the mechanisms leading to inv-dup del involve the formation of a dicentric chromosome which subsequently breaks to form monocentric duplicated and deleted chromosomes. Even for this case two different mechanisms may be considered to explain the onset of both different derivatives observed (Fig. 4, mechanisms 1 and 2). The absence in our case of the normal cell line argues in favour of a meiotic or very early post-zygotic dicentric onset through a deletion-induced repair mechanism, with meiotic or post-zygotic dicentric breakage, leading to the inv-dup del translocation t(2;5) in the cytotrophoblast through stabilization of the inv-dup del(5) by capture of a large segment of 2p containing the telomeric sequence. The cryptic 5p15.33 distal deletion in the second cell line is not the complementary deletion from dicentric breakage, but the same deletion present in the derivative, so a second early breakage event with loss of the additional 2q segment and de novo telomere synthesis (Fig. 4, mechanism 1) may have segregated the less unbalanced chromosome 5 in the inner cell mass, representative of both extraembryonic mesoderm and fetus, as further evidence of the rescue of unfavourable chromosomal imbalances [34]. Even for this case the mechanism 1 presupposes that a breakage would occur independently twice at the very same position of the initial breakpoint, which may seems unlikely. A simpler alternative mechanism that relies on an early post-zygotic error followed by independent stabilization of the inv-dup del by telomere capture in one daughter cell and by neo-telomere formation in the other ones has to be considered (Fig. 4, mechanism 2). Further derivatives from breakage and repair of the unstable dicentric, that have been lost during cell selection because of too large imbalances or that have been overlooked because not present in CV preparations, cannot be excluded.

Termination of pregnancy in both case 3 and case 4 did not allow the analysis of fetal tissues, although the selection towards the embryo of the smaller imbalances and, for case 4, a further post-zygotic rescue with loss of the duplicated segment, resulting in a simple terminal deletion 5p, may not be unlikely, as shown in our previous report [22]. The involvement of chromosomes 2 (as for case 3) and 5 in our case is in line with the description that certain chromosomes, i.e. 1, 2, 5, 16 and 18, are prone to cause constitutional complex chromosome rearrangements [10, 11].

In conclusion, our new four cases displaying mosaic derivative chromosomes during prenatal diagnosis add valuable clues for understanding the mechanisms underlying de novo structural rearrangements, by documenting the presence of unstable intermediate gross imbalances and their further breakage events and by showing the ‘backstage’ of the ongoing process in favour of the smallest imbalances, which are finally able to circumvent the early embryo selection.

## Data Availability

No datasets were generated or analysed during the current study.
